# The Role of Atypical Sexual Preference and Behavior in Neuroelectrophysiological Research of Human Sexual Behavior

**DOI:** 10.1007/s10508-021-01927-8

**Published:** 2021-02-11

**Authors:** Inka Ristow, Christian Kärgel

**Affiliations:** 1 Clinical Affective Neuroimaging Laboratory, Department of Psychiatry and Psychotherapy, Jena University Hospital, Jena, Germany; 2 Department of Diagnostic and Interventional Radiology and Nuclear Medicine, University Medical Center Hamburg-Eppendorf, Martinistraße 52, 20246 Hamburg, Germany; 3 Division of Forensic Psychiatry, LWL-University Hospital, Ruhr University Bochum, Bochum, Germany

We have read, with great interest, the Target Article by Ziogas, Habermeyer, Santtila, Poeppl, and Mokros (2020). It provides a unique and extensive overview of neuroelectrophysiological research related to a broad range of human sexual behavior. In their systematic review, Ziogas et al. covered more than eight decades of research represented in 255 studies of electric recording (including event-related potentials, somatosensory evoked potentials, quantitative electroencephalography, source reconstruction, and magnetoencephalography) and stimulation (including deep brain stimulation, electroconvulsive therapy, transcranial magnetic stimulation, and transcranial direct current stimulation). Among the range of analyzed brain potentials during sexual stimulation, P3 and late positive potential (LPP) showed significant effect sizes, and across all included studies, Ziogas et al. conclude a global potential of neuroelectric methods to distinguish sexual arousal from other states of emotional experiences.

However, since Ziogas et al. (2020) largely researched neuroelectric correlates of sexual arousal in teleiophilic (sexually attracted to adults) homosexual and heterosexual adult women and men, but also addressed populations with “abnormal” sexual preferences, the question arises as to whether their global findings can be generalized to more specific psychiatric populations. As Ziogas et al. correctly stated, only a few studies assessed the subject of neuroelectric recording in populations with atypical sexual preferences. In the context of this invited commentary as well as from a forensic psychiatric perspective, we would therefore like to take the opportunity to address certain methodological aspects that we believe should be considered for future investigations in this field.

Atypical sexual preferences, per se, do not automatically lead to psychological strain or criminal behavior but persons at risk have an increased propensity for both. For instance, pedophilia has been identified as a key risk factor for child sexual offending (CSO) and studies suggested that about 40–50% of juridical recorded cases of CSO are committed by pedophilic men (Seto, 2018). Not least due to the large number of estimated victims or the severe and long-term psychiatric consequences (including post-traumatic stress disorder, other anxiety disorders, depression, or suicidal behavior), research targeting the neurobiology of pedophilia and CSO is of explicitly high relevance. In order to protect potential future victims and reduce the amount of recidivism, the development of effective clinical intervention strategies for affected persons is essential, which demands a better understanding of the neurobiological basis underlying pedophilia and CSO.

Accordingly, using neuroscientific methods both phenomena have been investigated with increasing interest during the last years, particularly using (functional) magnetic resonance imaging ([f]MRI). Even with this increasing popular method, however, studies of neuroelectric correlates have been very underrepresented. This is most likely explainable on the basis of several barriers that relate to the feasibility of study assessment. For example, as Ziogas et al. (2020) mentioned, recruitment of appropriate study populations represents one of the major concerns. This concern is also reflected by methodological protocols in most of the published studies, which have only compared mixed and small groups of pedophilic offenders and non-offenders with healthy or forensic controls. It is important to note that pedophilia as an atypical sexual preference and CSO as a component of sexually delinquent behavior are regarded as different clinical entities, which has been corroborated by several recent neuroscientific investigations (Kärgel et al., 2015, 2017; Kruger et al., 2019; Massau et al., 2017; Schiffer et al., 2017).

Within these populations, the use of neuroelectric methods may be highly relevant in the investigation of potential biomarkers. While previous single studies reported encouraging findings associated with atypical sexuality across different domains including brain structure (Abé et al., 2020; Lett et al., 2018), epigenetics (Kruger et al., 2019), brain function (Cantor et al., 2016), and executive functions (Massau et al., 2017), these results await to be confirmed. Accordingly, in their review, Jordan, Wild, Fromberger, Müller, and Müller (2020) stated that none of the existing approaches meet the criteria of a clinical applicable diagnostic, response, or predictive biomarker for pedophilia or CSO. Therefore, in contrast to the application of a single marker, the combination of several variables is suggested to be the most promising approach, and the incorporation of unexplored methods might help to draw a more precise picture of the phenomenon. For example, behavioral studies reported abnormal cognitive performance in pedophilic child sexual offenders (Eastvold, Suchy, & Strassberg, 2011; Massau et al., 2017; Schiffer & Vonlaufen, 2011). Consequently, inclusion of neuroelectric parameters as correlates of cognitive performance could be beneficial in paradigms assessing sexual salience processing and therefore might contribute to a composite biomarker score.

The Target Article by Ziogas et al. (2020) also points to the absence of magnetencephalography (MEG) studies in the neuroelectric assessment of atypical sexuality, which allow for both high temporal and spatial resolution of neuroelectric responses, as compared to fMRI or electroencephalography (EEG). A recent study by Krylova et al. (2020) that was published after the release of the Ziogas et al. was the first to identify MEG correlates of sexual stimulus processing in the context of atypical sexual preference. Krylova et al. used sexual pictures of adults and children in a visual oddball paradigm and investigated the magnetic visual mismatch negativity event-related field response. Different neurophysiological signatures in both preconscious and later event-related field components were found in 17 pedophilic child sexual offenders and 20 healthy control subjects. While the use of MEG in this field of research is unique and a promising approach, the study also had some restrictions that have already been discussed above. Among them, only pedophilic child sexual offenders were recruited, and these individuals differed significantly in IQ compared to healthy control subjects. Future ERP-studies, especially those that aim to collect parameters of cognitive performance measures, such as reaction times or response accuracy, should control for IQ and cognitive function across groups to rule out potential confounds.

A further shortcoming of existing neuroelectric studies in abnormal sexual preferences is related to the presentation of appropriate visual stimulus material. As also pointed out by Ziogas et al. (2020), ideally neuroelectric recordings should be preceded by a validation of applied visual material. Validation should involve valence and arousal ratings by the individuals taking part in the experiment. This would also control for potential confounds due to participants who are sexually attracted to both children and adults (pedo-teleiophilic). Prevalence estimates ranging from 7 to 34% show that an exclusive sexual attraction toward children is rather rare (Bailey, Hsu, & Bernhard, 2016; Beier et al., 2015).

Another methodological approach which has been largely neglected in the investigation of atypical sexuality is the assessment of electrical stimulation, which could, in principle, open up a potential for the development of therapeutic interventions. For example, impulsive behavior has been described as a predictor of sexual recidivism (Hanson & Morton-Bourgon, 2004), but recent studies point to the association between specific executive dysfunctions and an increased probability of engaging in sexual offending behavior (e.g., Eastvold et al., 2011; Massau et al., 2017; Ristow et al., 2018). Accordingly, the investigation and electrophysiological treatment of structures related to specific cognitive (dys)functions (i.e., inhibitory control or impulsivity) may be of great interest as target regions for intervention. To our knowledge, no study has been published until the publication date of the Ziogas et al. (2020) article, where transcranial direct current stimulation (tDCS) or transcranial magnetic stimulation (TMS) was applied in patients with sexual preference disorders. However, there is a preregistered study by Pezzoli et al. (2020) which was published after the release of Ziogas et al. In the Pezzoli et al. study, automatic attention bias for child versus adult visual stimuli was investigated in 16 pedophilic child sexual offenders and matched healthy control subjects while receiving a tDCS over the left dorsolateral prefrontal cortex (dlPFC), a brain region that has been implicated in neural processes of cognitive control and sexual arousal (Poeppl et al., 2013; Walter et al., 2007). Although a differentiation between offense statuses in the pedophilic study group would have been very interesting for the exploration of potential longitudinal effects, electric stimulation might be a promising approach with close clinical implications.

The development of effective prevention strategies for future sexual delinquency may also benefit from the translation of the neurobiological knowledge underlying pedophilia and CSO into clinical and diagnostic approaches. For example, it is clinically essential to be able to clearly identify pedophiles with a sexual fixation toward prepubescent children (exclusive type) in contrast to those who are also attracted to adults (non-exclusive type), since from a therapeutic perspective, each sub-type needs to be treated in a unique manner.

Besides its potential advantage, however, the definition of biomarkers in the context of atypical sexual interests and behavior is not undisputed and entails the risk of a false positive rating, which may be associated with legal, but also social and intra-psychological consequences that can be serious on a case-by-case basis (Komisaruk, 2020). Taking such ethical considerations into account, it is important to rather treat potential biomarkers for sexual interests as additional tools but not like single indicators for diagnostic, clinical or risk assessment purposes.

Despite the great amount of attention and media coverage that is granted to this sensitive topic, there is an obvious gap in terms of our knowledge of neuroelectric correlates of atypical sexuality which is revealed by the Ziogas et al. (2020) Target Article. Gaining a deeper understanding of the neurophysiological correlates of atypical sexual preferences, such as pedophilia, is essential to optimize prevention and rehabilitation programs for child sex offenders. Therefore, it would be desirable if future research in the field of neuroelectric research of human sexual behavior would also address the role of atypical sexual preference and behavior.
